# Robust and Elastic Lunar and Martian Structures from 3D-Printed Regolith Inks

**DOI:** 10.1038/srep44931

**Published:** 2017-03-20

**Authors:** Adam E. Jakus, Katie D. Koube, Nicholas R. Geisendorfer, Ramille N. Shah

**Affiliations:** 1 Department of Materials Science and Engineering, McCormick School of Engineering and Applied Science, Northwestern University, 2220 Campus Drive, Room 2036, Evanston, IL 60208, USA; 2 Simpson Querrey Institute for BioNanotechnology, Northwestern University, 303 E Superior St., 11^th^ Floor, Chicago, IL 60611, USA; 3 Department of Biomedical Engineering, McCormick School of Engineering and Applied Science, 2145 Sheridan Road, Evanston, IL 60208, USA; 4 Department of Surgery – Organ Transplantation Division Comprehensive Transplant Center, Feinberg School of Medicine, Northwestern University, 676 N. Saint Clair St, 19^th^ Floor, Chicago, IL 60611, USA.

## Abstract

Here, we present a comprehensive approach for creating robust, elastic, designer Lunar and Martian regolith simulant (LRS and MRS, respectively) architectures using ambient condition, extrusion-based 3D-printing of regolith simulant inks. The LRS and MRS powders are characterized by distinct, highly inhomogeneous morphologies and sizes, where LRS powder particles are highly irregular and jagged and MRS powder particles are rough, but primarily rounded. The inks are synthesized via simple mixing of evaporant, surfactant, and plasticizer solvents, polylactic-co-glycolic acid (30% by solids volume), and regolith simulant powders (70% by solids volume). Both LRS and MRS inks exhibit similar rheological and 3D-printing characteristics, and can be 3D-printed at linear deposition rates of 1–150 mm/s using 300 μm to 1.4 cm-diameter nozzles. The resulting LRS and MRS 3D-printed materials exhibit similar, but distinct internal and external microstructures and material porosity (~20–40%). These microstructures contribute to the rubber-like quasi-static and cyclic mechanical properties of both materials, with young’s moduli ranging from 1.8 to 13.2 MPa and extension to failure exceeding 250% over a range of strain rates (10^–1^−10^2^ min^−1^). Finally, we discuss the potential for LRS and MRS ink components to be reclaimed and recycled, as well as be synthesized in resource-limited, extraterrestrial environments.

Establishing autonomous or inhabited extraterrestrial sites has been part of science fiction culture for many years. This past decade has witnessed not only tremendous advances in technology that can make such endeavors technically feasible, but also substantial growth in commercial and government interest. Developing the capacity to establish and maintain extraterrestrial sites on the Moon, Mars, and additional planetary and large, non-planetary bodies would be an achievement for humanity and would lay the foundation for further, extraterrestrial scientific and engineering advances as well as private commercialization. To this end, additive manufacturing (AM) and 3D-printing (3DP) approaches have recently been considered as promising means to enable prolonged off-world activities through utilization of native planetary regoliths for manufacturing^1^,^2^,^3^,^4^,^5^. Although promising, current AM and 3DP approaches, such as those that utilize powder beds, high-energy beams, or both to selectively sinter or melt regolith materials suffer from numerous process- and material-related restrictions^1^,^6^ that make them ill-suited for utilization in such environmentally extreme, resource-starved, reduced-gravity environments. On the other hand, while traditional material-deposition 3D-printing approaches, such as fused deposition modeling (FDM), are currently being successfully and safely utilized in the micro-gravity environment of the International Space Station (Zero-G Printer, Made In Space) to create objects on demand, traditional deposition approaches have only been compatible with a select set of simple thermoplastics and low-particle-content thermoplastic composites, but not regolith materials. Additionally, although valuable for a variety of applications, previous work with planetary regoliths has focused entirely on the fabrication of hard materials, primarily via thermal^1^,^3^,^4^ or microwave sintering^4^,^7^, melting of regolith powder compacts, or cementation reactions of extruded materials^2^, and has not addressed the need for soft-material manufacturing.

In this work, we extend a 3D-printable particle-based liquid ink platform that we have previously utilized to create and 3D-print metals and alloys^8^,^9^ as well as regenerative ceramic and graphene biomaterials^10^,^11^,^12^ (from relatively, compositionally and morphologically pristine powders) to Lunar^13^ and Martian^14^ regolith simulants (LRS and MRS; JSC-1A and JSC MARS-1A, respectively); which are unrefined, highly compositionally and morphologically inhomogeneous natural, volcanic materials that emulate the surface environments of specific regions on the Moon and Mars, respectively^15^,^16^. The results are rapidly 3D-printed, highly versatile and mechanically elastic composites comprised of approximately 90 wt. % LRS or MRS and 10 wt.% bio-derived polymer (polylactic-co-glycolic acid, PLGA) that have the potential to address the need for soft materials off-world.

## LRS and MRS Ink Synthesis and Characterization

**Figure 1:**
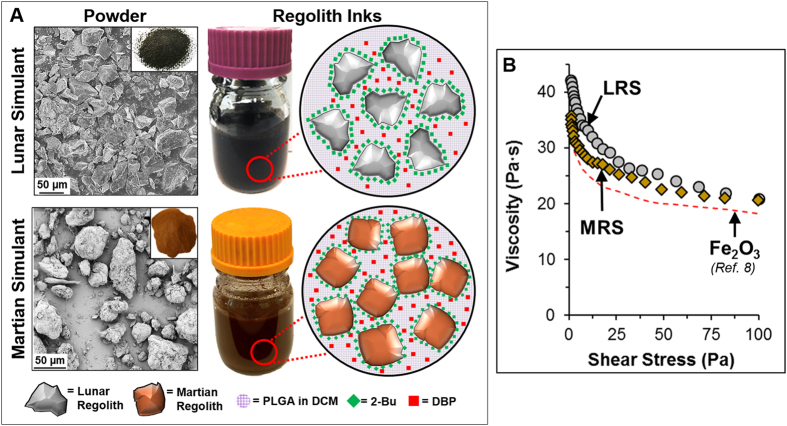
(**A**) Representation of the Lunar and Martian regolith simulant inks, LRS and MRS, respectively. On left, scanning electron micrographs and photographs (inset) of sieved JSC-1A lunar regolith simulant and MARS JSC-1A Martian regolith simulant powders. On right, Photographs of ~100 mL of LRS and MRS inks and schematic representations of the ink compositions and hypothesized, individual component distributions. (**B**) Viscosity as a function of shear stress of LRS and MRS inks. Viscosity as function of shear stress of previously reported 70 vol.% Fe_2_O_3_ inks^8^ shown for comparison.

Lunar and Martian regolith simulant (LRS) inks are synthesized according to processes we have described previously for metal and metal oxide, ceramic, graphene, and mixed particle ink systems^8^,^9^,^10^,^11^,^12^. The inks are comprised of three major components: powder, elastomeric binder, and a solvent mixture. The powders utilized for this work were commercially available, 325-mesh sieved JSC-1A Lunar and JSC MARS-1A regolith simulants. As received Lunar and Martian regolith simulant powders contained particles with diameters as large as 1 mm (Supplementary Figure 1). Sieving was necessary to remove these larger particles that would otherwise comprise the 3D-printability of the inks described below. Initial attempts were made to 3D-print inks created from as-received JSC-1A and JSC MARS-1A regolith powders, but these could not be extruded from nozzles less than 2 mm in diameter. The morphologies of the sieved LRS and MRS powders are shown in Fig. 1A, and nominal compositions can be found in Supplementary Table 1^15^,^16^. Despite being compositionally similar; comprised primarily of silicates, aluminates, and iron oxides, LRS and MRS powders are poly-disperse (several nanometers to >50 μm) and morphologically distinct, exhibiting irregular jagged contours on the LRS powders and rough, but rounded contours on MRS powders. The elastomeric binder was commercially available polylactide-co-glycolide (PLGA: Evonik), a commonly utilized medical polymer^17^,^18^ that can be synthesized from biologically derived, renewable reagents^19^,^20^, and which we have published on previously in relation to the 3D-printing ink system^8^,^9^,^10^,^11^,^12^. Regolith powder and PLGA are incorporated into the inks in (70–75% by volume regolith, 30–25% by volume PLGA), which corresponds to approximately 85 wt.% LRS and MRS powder (solids content only). The solvent mixture that has also been described in detail previously^8^,^9^,^11^,^21^, includes majority dichloromethane (DCM), a high volatility evaporant, and less quantities of 2-butoxyethanol (2-Bu), a non-specific surfactant, which mitigates electrostatic and steric interactions between suspended particles, and dibutyl phthalate (DBP), a plasticizer that improves the flow properties of the dissolved PLGA and further inhibits particle-particle interaction during flow. After thickening via evaporation of excess DCM to a printable consistency, both LRS and MRS exhibit similar rheological, shear-thinning characteristics (Fig. 1B), that are also similar to previously described metal and ceramic inks synthesized using this approach^9^. This indicates that ink synthesis and resulting rheological properties are primarily independent of particle composition and morphology. Also similar to previously reported inks^9^,^10^,^11^, LRS and MRS inks are shelf-stable, and can be stored for at least several months prior to use.

## 3D-Printing LRS and MRS Inks

**Figure 2:**
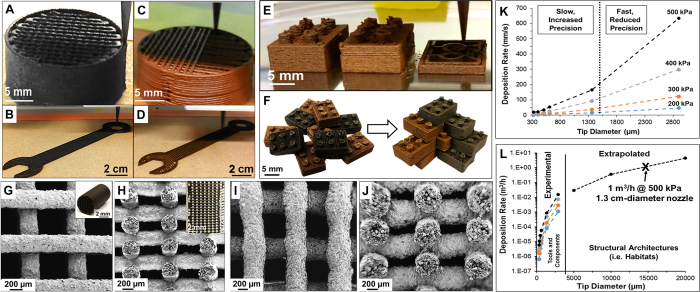
(**A–D)** Photographs of LRS (**A**,**B**) and MRS (**C**,**D**) inks being 3D-printed into a many-layered 2 cm-diameter cylinders and 12 cm long wrenches, respectively. (**E**) Photograph of MRS ink being 3D-printed into multiple stackable building blocks. (**F**) Photographs of 3D-printed LRS and MRS building blocks before and after manual assembly into an arbitrary structure. (**G**,**J**) SEM micrographs of 3D-printed LRS (**G**,**H**) and MRS (**I**,**J**) structures. Top-down (**G**,**I**) and cross-sectional (**H**,**J**) are shown. Insets show macroscopic photographs of structures. (**K**) Linear deposition (extrusion) rate of LRS Ink as a function of applied pressure and nozzle diameter. Low pressures and small tip diameters result in increased fidelity, but decreased fabrication times, while high pressures and larger tip diameters result in reduced fidelity, but faster fabrication times. (**L**) Experimentally measured and corresponding extrapolated volumetric deposition rates of LRS inks as a function of applied pressure and nozzle diameter. Colored lines correspond to extrusion pressures indicated in (**K**).

Despite being in liquid form, the LRS and MRS inks can be rapidly 3D-printed into user defined architectures (Fig. 2A,E; Supplementary Videos 1 and 2), at linear 3D-printing speeds demonstrated upwards of 150 mm/s, resulting in objects that do not require time to dry and can be handled immediately (Supplementary Video 3). LRS and MRS 3D-printing parameters and behavior do not significantly differ. Figure 2F illustrates this similarity through the production and assembly of LRS and MRS building blocks, which were 3D-printed from the same digital file using near identical 3D-printing parameters. The LRS and MRS structures maintain their as-printed fidelity (Fig. 2G–J), and successive layers of 3D-printed material can even span gaps (Fig. 2H,J), which is enabled by the rapid solidification of the material via DCM evaporation upon extrusion. It is important to note that adhesion of the first deposited layer of material to the platform substrate is important not only for ensuring a successful print-job on Earth (gravity = 1), but for ensuring success in reduced-gravity environments such as the Moon (gravity = 0.17) and Mars (gravity = 0.38). In this work, standard sand papers and silicon carbide papers (320grit) resulted in substantial adhesion of the first layer, and resulting printed object, to the platform while still permitting the object to be easily removed without sustaining damage or altering the underlying substrate (allowing the sandpapers and silicon carbide papers to be used repeatedly).

Importantly, the regolith inks are compatible with a range of nozzle diameters and extrusion pressures (Fig. 2K), imparting significant versatility and control to the user with respect to the types of structures that can created, achievable resolution (Supplementary Figure 2), and fabrication rate. On the finer end of the spectrum, LRS inks were able to be continuously be extruded from 330 μm nozzles without clogging at linear deposition rates as low as 2.1 mm/s (200 KPa) and as high as 19.2 mm/s (500 KPa). Similarly, 2.9 mm-diameter nozzles were utilized under the same low and elevated extrusion pressures to achieve linear deposition rates of 46 and 633 mm/s, respectively. MRS inks exhibit a similar rate v. pressure relationship, but were unable to be extruded from 330 μm nozzles without frequent clogging events, which was likely due to the presence of large particles (Fig. 1A). In terms of volume, this corresponds to 6.5 × 10^−7^ – 1.5 × 10^−2^ m^3^/h. This versatility provides a large, parametric space with which to create both small, high resolution objects, and large, low-resolution objects, without altering the ink. If the ink is further thickened, to a consistency similar to soft modeling clay, large extrusion diameters, such as 1.4 cm, can be achieved (Supplementary Figure 3), potentially resulting in single-nozzle volumetric deposition rates approaching 1 m^3^/h. Multi/parallel nozzle extrusion-based 3D-printing platforms^22^ could potentially be employed to substantially increase material deposition and fabrication rates, allowing for high-throughput production of small and moderate sized parts. Additionally, similar to the metal, metal oxide, ceramic, and graphene 3D systems described by Jakus *et al*.^8^,^10^,^11^,^12^. 3D-printed LRS and MRS objects can be recycled into new inks, by dissolving them in DCM, or joined to previously 3D-printed objects via application of ink at points of contact^9^,^10^,^11^. It is unclear at this time, however, what modifications would need to be made to the 3D-printing process to permit it to be successfully applied in external environments characterized by low atmospheric pressures and extreme temperatures. Regardless, the process in its current form can be readily employed in a pressurized moderate temperature environment.

## 3D-Printed LRS and MRS Microstructures

**Figure 3:**
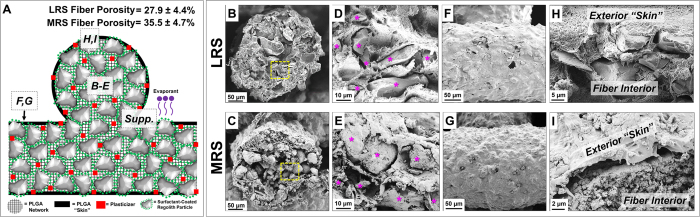
(**A**) Schematic representation of the composition and structure of multi-layered 3D-printed LRSor MRS (symbols and colors consistent with those portrayed in the ink schematic in Fig. 1). Regolith particle represents both Lunar and Martian Particles. Letters enclosed in dashed boxes correspond to regions from which SEM images B-I, and Supplementary Figure 4D–G were obtained. Average porosity of LRS and MRS fibers is indicated, and porosity as function of fiber diameter can be found in Supplemental Figure 4. (**B,C**) SEM micrographs of single LRS and MRS fiber cross-sections. (**D,E**) Higher magnification micrographs of area indicated (yellow box) in B and C, respectively. Magenta “*” indicate LRS or MRS powder particles. (**F,G**) SEM micrographs of the surface of an extruded LRS and MRS fiber within a 3D-printed object. (**H,I**) SEM micrographs of the cross-sections of LRS and MRS fibers highlighting the exterior PLGA “skins” adjacent to the particle-rich interiors.

Closer inspection of 3D-printed LRS and MRS structures (Fig. 3), reveals distinct internal and external microstructures, schematically illustrated in Fig. 3A. The interior of each material is defined by a spatially tight distribution of LRS or MRS particles connected, and in some cases, physically encapsulated, continuous PLGA elastomer networks (Fig. 3B–E; Supplementary Figure 4B,C). Density measurements of extruded LRS and MRS fibers indicate material porosity to be 27.9 ± 4.4% and 35.5 ± 4.7%, respectively, which appears to be independent of fiber (extruding nozzle) diameter for nozzles ranging in size from 330 to 2900 μm (Supplementary Figure 4A). The exterior of the materials are characterized by a minimally porous, thin (< 5 μm) PLGA “skin” connecting periodically erupting particles (Fig. 3F–I). The PLGA skin arises from the volumetrically, inhomogeneous evaporation of DCM, and subsequent PLGA precipitation, throughout the volume of the extruded fibers. Due to the surface of the extruded material being exposed to atmosphere, the DCM rapidly evaporates, creating a near-solid PLGA layer, which is analogous to “skins” that develop on the surfaces of common paints which have been exposed to air for extended periods of time. Despite the rapid-drying nature of the deposited ink, the DCM present in freshly extruded ink locally dissolves the PLGA within the surface of the previously deposited material with which it comes into contact^11^. This results in physically, near-seamless transitions and continuity between individual layers as well as adjacent, contacting material in the same layer (Supplementary Figure 4D–G). This rapid solidification and bonding to previously deposited layers has important implications for successful use in reduced-gravity environments, where liquid drift or poor-adhesion between deposited layers would otherwise yield poor results.

We hypothesize that this inhomogeneous drying and the resulting elastomer skin, although thin, is the primary characteristic that permits 3D-printed LRS objects to be handled immediately after 3D-printing, and also imparts unique mechanical properties to the material (discussed later) despite the material being comprised primarily of rigid, irregular powder particles. The characteristic microstructures of the 3D-printed LRS and MRS enable their manipulation and post-3D-printing processing in numerous, advantageous ways, including “soft-polishing” via solvent smoothing (Supplementary Figure 5A) and cutting (Supplementary Figure 5B–E).

## 3D-Printed LRS and MRS Mechanical Properties

**Figure 4:**
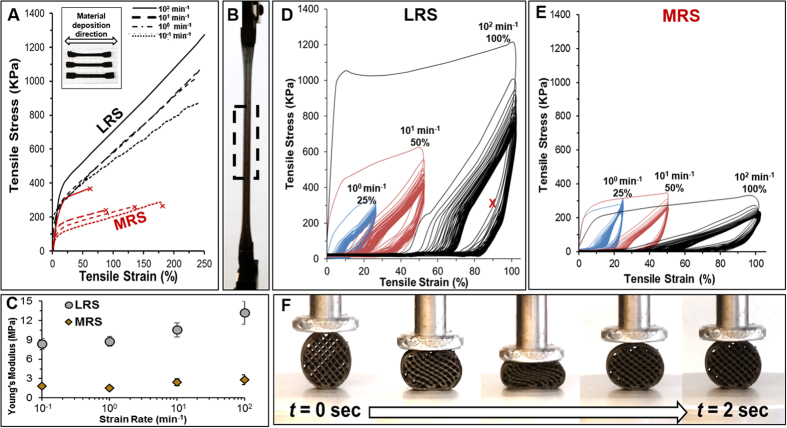
(**A**) Representative tensile curves of 3D-printed LRS and MRS tensile specimens loaded at indicated strain rates. Tensile tests were performed until fracture on MRS samples and 250% strain on LRS samples, which did not fracture. (**B**) Photograph of LRS tensile specimen at approximately 250% strain. Black-dashed box indicates gauge length at strain of 0%. (**C**) Young’s modulus as a function of strain rate for LRS and MRS tensile specimens. (**D**) The first 100 cycles of LRS and (**E**) MRS tensile samples extended at indicated strain rates to 25, 50, and 100% strain. (**E**) Time-series photographs of a 1 cm-diameter, 0–90° patterned cylinder, loaded at 45° undergoing a single compression cycle (10^2^ min^−1^ strain rate, 50% strain).

3D-printed LRS and MRS can undergo substantial tensile deformation (Fig. 4A,B) prior to failure, including 15–20% elastic strain, across a range of strain rates (10^−1^ min^−1^ – 10^2^ min^−1^). MRS objects failed between 50–175% strain, while LRS objects were strained upwards of 250% without failing. 3D-Printed LRS and MRS exhibit young’s moduli of ~8–13 MPA, and ~2–3 MPa, respectively, depending on strain rate (Fig. 4C). When extended to cyclic tensile deformation (Fig. 4D,E), both 3D-printed LRS and MRS can be repetitively deformed at slow (1 min^−1^) and rapid (10^2^ min^−1^) rates, up to 100% and not fracture or substantially fatigue. However, the 3D-printed objects are not able to recover to full net shape if strained beyond 15–20%. Similar elastic properties can be observed in 3D-printed objects undergoing cyclic compression (Fig. 4F), but it must be noted that compressive properties, unlike tensile properties of dense dog-bone tensile specimens, are heavily dependent on the 3D-printed geometry and porosity, as well as on the loading configuration.

These mechanical results indicate that the mechanical properties of the 3D-printed materials are dominated by the continuous PLGA matrix, rather than the rigid LRS and MRS particles, and are in agreement with previously described mechanical properties and deformation mechanisms of materials made from 3D-printed hydroxyapatite inks^10^, synthesized using the same process used here. Like previously described systems, these mechanical properties permit the as-printed LRS and MRS materials to be deformed in a variety of ways, including rolling and folding (Supplementary Figure 6).

## Component Collection and Recycling

**Figure 5:**
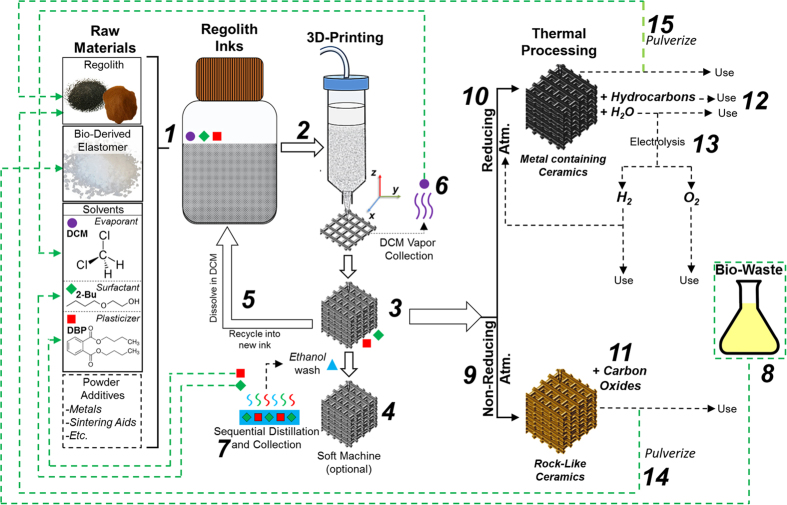
Schematic representation of ink components, LRS and MRS ink synthesis *(1*), 3D-printing *(2),* final object (*3*), optional soft-machining (*4*), object recycling (*5*), and solvent collection and recycling (*6,7*). Because the polymer, PLGA, can be synthesized from biologically derived lactic and glycolic acid, it would be possible to use to process and recycle unrelated bio-wastes (*8*; urine, compost, etc.) into PLGA and similar bio-derived elastomers. Optionally, the 3D-printed elastic structures could potentially be transformed into rigid architectures via sintering in a non-reducing (*9*) or reducing (*10*) atmosphere, which would yield carbonaceous gases (*11*) and water/hydrocarbons (*12*), respectively, which could be further processed into diatomic oxygen and hydrogen via electrolytic methods (*13*). Finally, sintered regolith structures could be pulverized (*14,15*) to yield regolith powders, which could be utilized to make new regolith inks for 3D-printing.

For manufacturing approaches, additive or not, to be practical in several limited supply environments, such as the Moon or Mars, they must not only be versatile with regards to what they can create (structures and materials), but they must also be compatible with *in situ* resources and have the potential to be recycled as needed. Additionally, it is important that the manufacturing approach, if it is being performed in confined spaces with personnel (such as a Martian or Lunar habitat) either not yield harmful byproducts or be controlled such that harmful byproducts can be directed, filtered, and collected. Figure 5 shows a summary of the processes and properties of 3D-printed LRS and MRS objects, along with potential recycling and byproduct collection routes. Raw, unrefined (or refined in the case of metallic and other materials^5^) regolith powders can be collected directly from the extraterrestrial surfaces and sieved as needed. The elastomer can be synthesized from monomeric components, such as poly-lactic acid and poly-glycolic acid, isolated from biological sources such as compost and human urine^23^,^24^. The majority solvent used for 3D-ink synthesis, DCM, does not play a chemical role in the ink or 3D-printing process, and immediately evaporates upon deposition of material. Inhalation of small concentrations of DCM vapor, although not acutely toxic, could result in serious adverse health conditions over time. Because of this, it would be vital for the residual DCM vapors to be immediately exhausted out of the habit or filtered and quarantined, similar to the operation of the environmental controls developed for and used on the Zero-G Printer currently operating on the ISS that collects toxic nano-particulate vapors that result from the FDM process. The DCM vapor could also be potentially consolidated and collected using simple cold-condensation approaches and reserved for future use. Additionally, DCM can be synthesized directly through thermal (possibly solar driven) reaction of chlorine gas and methane, a biological waste product. Both 2-Bu and DBP can be removed from the 3D-printed materials via ethanol washing^10^. Due to the large variance in boiling temperatures (i.e. vapor pressures) of ethanol, 2-Bu, and DBP^11^, the resulting wash solutions could be sequentially distilled to separate and collect the three component solvents for recycled use. Finally, 3D-printed LRS and MRS objects could be sintered in reducing or non-reducing atmospheres to create rigid architectures, that could be used for a variety of structural applications where elasticity and mechanical flexibility is not required or may be at a disadvantage. These sintered structures could also be recycled via pulverization into powders for regolith inks.

## Conclusions

This new approach for additively manufacturing planetary materials represents a new, powder-bed-free and energy-beam-free, resource utilization scheme for fabricating user-defined, soft-material structures from unrefined, highly inhomogeneous regoliths. Processes for regolith ink synthesis appear to be primarily independent of regolith composition and particle morphology. Due to the purely physical nature of ink synthesis (simple mixing of components, with no chemical reactions/transformations involved this process is potentially scalable. Additionally, the simple, rapid extrusion nature of the regolith ink 3D-printing process is also likely amenable to application with parallel nozzle extruders, larger diameter extruders, large build-area platforms, mobile extruders, or any combinations thereof. Although not investigated in this work, the resulting printed structures (due to the high LRS and MRS particle packing densities) can also be processed to create hard regolith structures via post-3D-printing sintering, as described previously for 3D-printed metal, alloy, and metal oxide ink systems^8^,^9^. Collectively, this work represents an extension of a newly established, materials-centric 3D-printing platform to unrefined, physically and compositionally inhomogeneous powders, and also illustrates that soft-materials, can be fabricated from hard, inhomogeneous, native resources and components that can potentially be recycled and reused.

## Materials and Methods

Lunar and Martian Regolith inks were synthesized following previously established and published protocols for other particle-laden inks^8^,^9^,^10^,^11^,^12^. In brief, inks were synthesized by combining JSC-1A bulk lunar mare regolith simulant (sieved to < 50 μm, Orbitec), polylactic-co-glycolic acid copolymer (PLGA; 82:18 by weight polylactic acid:polyglycolic acid; Evonik Industries, USA) dissolved in dichloromethane (DCM; Sigma), 2-butoxyethanol (2-Bu; Sigma), and dibutyl phthalate (DBP; Sigma). For every 3.53 g of LRS powder (equivalent to 1 cm^3^) utilized to synthesize ink, 0.49 g PLGA (*ρ = *1.15 g/cm^3^) dissolved in ~5 mL DCM, 0.9 g 2-Bu, 0.45 g DBP, and an additional ~5 mL DCM were used, resulting in an ink containing a solids loading of 70 vol.% LRS powder and 30 vol.% PLGA. The resulting mixture was physically stirred, periodically, in an open container to permit excess DCM to evaporate. The resulting inks were rheologically characterized using previously described processes and conditions^8^,^10^. Martian regolith simulant (MRS) inks were synthesized using an analogous process, but substituting 3.5 g JSC MARS-1A (sieved to < 50 μm, Orbitec) in place of the 3.53 g/cm^3^ LRS powder. LRS and MRS inks were immediately 3D-printed or stored in air-tight containers at 4 °C until needed for up to 6 months.

All LRS and MRS samples were 3D-printed via direct extrusion, under ambient conditions using a 3D BioPlotter (EnvisionTec). Linear deposition speeds and extrusion pressures ranged from 5–120 mm/s and 100–550 KPa, respectively, depending on the samples produced. LRS and MRS samples intended for quasi-static and cyclic tensile testing were 3D-printed into a standard dog-bone geometry defined as being 20 mm long, 3 mm wide, and 1.5 mm thick using a 510 μm nozzle. The LRS ink was deposited in the direction of gauge-length (loading direction), with 450 μm spacing between parallel struts (resulting in a non-porous architecture), for a total of 5-layers (~1.5 mm thick samples). 12 × 12 cm LRS and MRS sheets were produced using a 600 μm diameter nozzle and deposition speeds of 60–80 mm/s. Each sheet was comprised of three-layers, each layer oriented 120° with respect to the previous layer.

LRS and MRS fiber porosities as a function of nozzle diameter were determined according to previously described methods^9^,^12^. In brief, extruded LRS and MRS fibers of varying diameter and approximately 0.5 m in length were collected, washed in ethanol to remove residual solvents, and dried overnight at 40 °C. The diameters and lengths of the dried fibers were measured along with their mass. Based on these values, actual density of the material was determined and normalized to the theoretical solid density (30% PLGA and 70% LRS or MRS), yielding percent porosity of the material. N = 3 was used for each material type and fiber diameter.

LRS and MRS objects were sectioned by hand using a razor blade or surgical scalpel after being frozen at −80 °C. LRS and MRS cylinders were “soft polished” by gently applying DCM solvent to the exterior contours using a standard paint brush, and brushing perpendicular (along the cylinder length) to the ridges.

Scanning electron microscopy was performed using a LEO Gemini 1525 SEM after samples had been coated in 15 nm of osmium via osmium plasma.

All mechanical testing and measurements for as-printed LRS samples were performed by and obtained using a LF Plus mechanical tester (Lloyed Instruments) equipped with a 50 N load cell. Tensile specimens (described in 3D-printing section of materials and methods) were quasi-statically loaded at strain rates of 0.1, 1, 10, and 100 min^−1^ (n = 3 for each strain rate) until 250% strain (50 mm displacement) had been attained. Cyclic tension tests were performed on identically 3D-printed LRS and MRS tensile specimens under the following conditions: 1 min^−1^ to 25% max strain, return to 0% strain, for 500 cycles; 10 min^−1^ to 50% max strain, return to 0% strain, for 500 cycles; 100 min^−1^ to 100% max strain, return to 0% strain, for 500 cycles. No fracture or macroscopic taring was observed in any of the samples.

## Additional Information

**How to cite this article:** Jakus, A. E. *et al*. Robust and Elastic Lunar and Martian Structures from 3D-Printed Regolith Inks. *Sci. Rep.* **7**, 44931; doi: 10.1038/srep44931 (2017).

**Publisher's note:** Springer Nature remains neutral with regard to jurisdictional claims in published maps and institutional affiliations.

## Supplementary Material

Supplementary Information

Supplementary Dataset 1

Supplementary video 1

Supplementary video 2

Supplementary video 3
