# Supplementary material for: Robust and Elastic Lunar and Martian Structures from 3D-Printed Regolith Inks
Source: Sci Rep. 2017 Mar 20;7:44931. doi: 10.1038/srep44931 (PMC5357966; doi:10.1038/srep44931)
Supplement: Supplementary Information [file srep44931-s1.pdf]

Robust and Elastic Lunar and Martian Structures from 3D-Printed Regolith Inks

*Adam E. Jakus[1,4], Katie D. Koube[1,4], Nicholas R. Geisendorfer[1,4], and Ramille N. Shah[1,2,3,4]**

[1]Department of Materials Science and Engineering, McCormick School of Engineering, Northwestern University
[2]Department of Biomedical Engineering, McCormick School of Engineering, Northwestern University
[3]Divsion of Organ Transplantation, Comprehensive Transplant Center, Department of Surgery, Feinberg School of Medicine, Northwestern University
[4]Simpson Querrey Institute for BioNanotechnology, Northwestern University

Dr. Adam E. Jakus, PhD
303 E Superior St.
11th Floor, SQI
Chicago, IL 60611 USA

Katie D. Koube
303 E Superior St.
11th Floor, SQI
Chicago, IL 60611 USA

Nicholas R. Geisendorfer
303 E. Superior St.
11th Floor, SQI
Chicago, IL 60611 USA

*Prof. Ramille N. Shah, PhD
303 E. Superior St.
11th Floor, SQI
Chicago, IL 60611 USA
E-mail: ramille-shah@northwestern.edu

Supplementary Materials and Methods

Additional LRS and MRS structures, such as wrenches, building blocks, etc. were produced from open-source .STL geometries (thingiverse.com) using 330-1200 µm nozzles and a range of printing speeds and extrusion pressures. Linear deposition rates for LRS inks were measured as a function of nozzle diameter (330 µm, 410 µm, 510 µm, 610 µm, 1360 µm, 2900 µm; Nordson EFD). The 2900 µm diameter nozzle was adapted from a 1360 µm conical nozzle by cutting the nozzle tip, resulting in a 2900 µm opening. Rates were also measured as a function of applied extrusion pressures. For a given nozzle diameter and extrusion pressure, pressure was applied via the 3D BioPlotter for exactly five seconds. The lengths of the resulting extruded fibers were measured and normalized to time. Volumetric deposition rates were calculated from linear deposition rates, taking into account the cross-sectional areas and total volume of extruded fibers. A power-series fit (R=0.98) was applied to the experimental volumetric deposition-rate date to extrapolate possible volumetric deposition rates for larger diameter nozzles.

Supplementary Tables

Table 1. Nominal compositions (wt. %) of LRS (JSC-1A) and MRS (JSC MARS-1A) regolith powders as reported by corresponding materials data sheets (Orbitec LLC).

	SiO_2	Al_2O3	Fe_2O_3	FeO	TiO_2	CaO	Na_2O
**JSC-1A**	46	15.75	12.2	8.17	1.7	9.9	2.8
**JSC MARS-1A**	40	22	11	3	3.5	5.5	2

Supplementary Figures

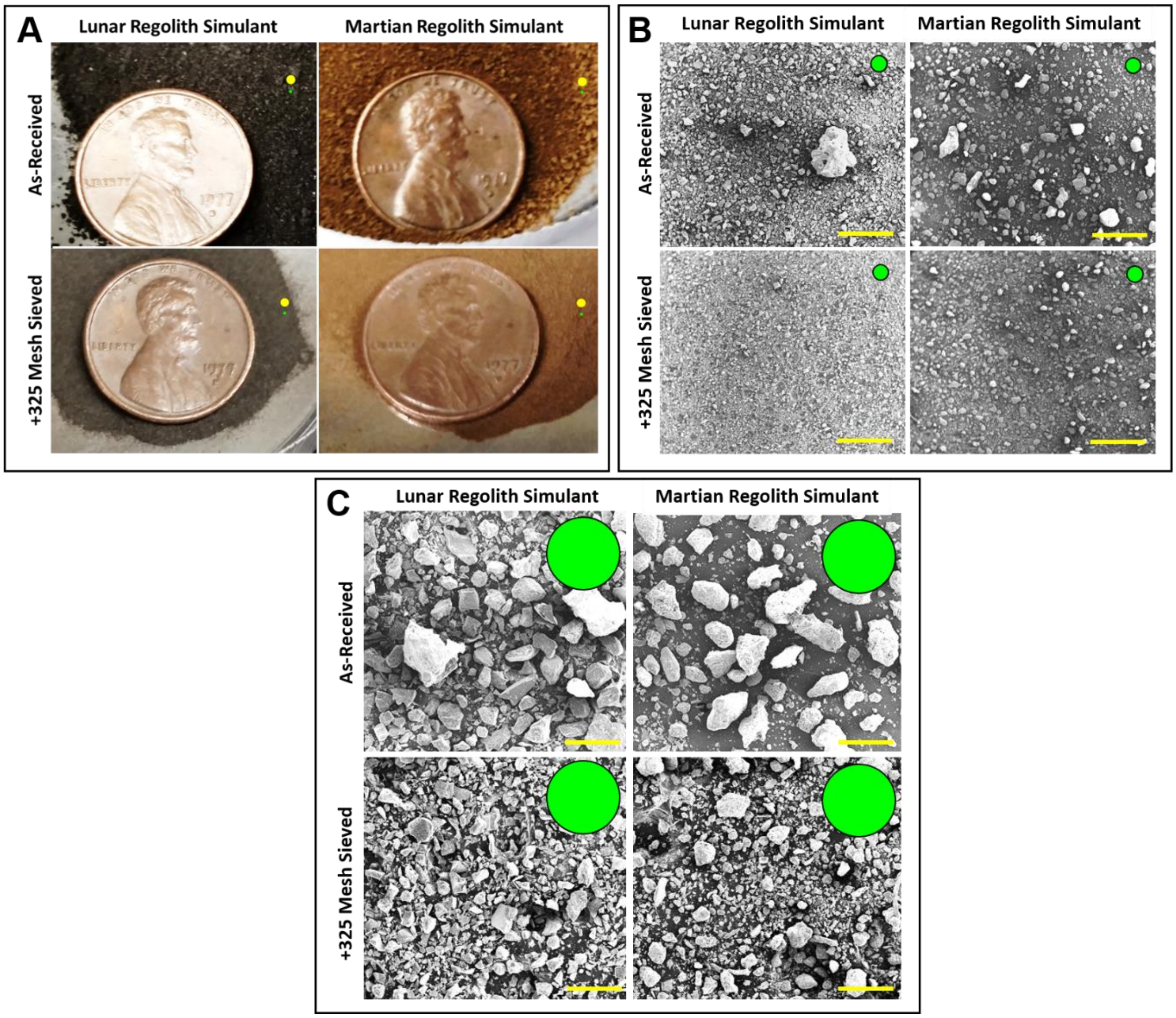

Supplementary Figure 1. A) Photographs of LRS and MRS powders, as received and after undergoing +325 mesh sieving. United States penny included for reference. Yellow and green dots represent 1 mm and 330 µm, respectively. 330 µm was the finest nozzle diameter found to be able to effectively extrude LRS and MRS inks derived from sieved powders without frequent clogging events. **B)** Scanning electron micrographs of LRS and MRS powders, as received and after undergoing +325 mesh sieving. Scale bars are 1 mm, and green dot is 330 µm in diameter. **C)** Additional scanning electron micrographs of LRS and MRS powders before and after sieving. Scale bars are 250 µm and green dot represents 330 µm in diameter.

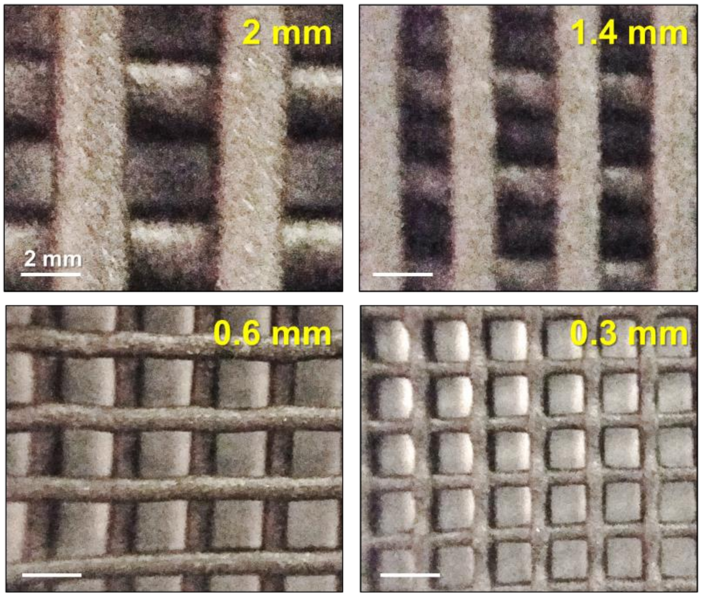

Supplementary Figure 2. Top-down view of LRS cylinders 3D-printed using indicated nozzle diameters. All photographs portrayed at the same scale.

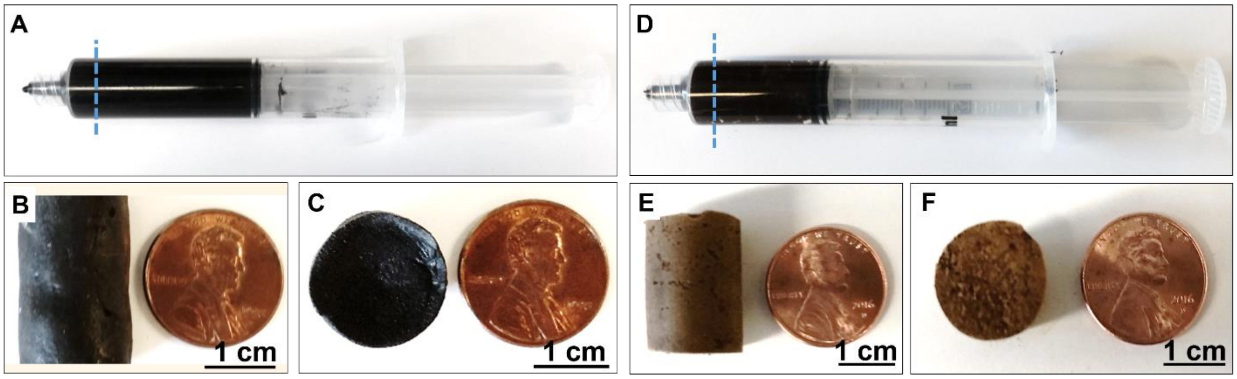

Supplementary Figure 3. A) Photograph of standard syringe loaded with LRS ink. Blue-dashed line indicated point at which the syringe was cut to result in a 1.4 cm-diameter orifice, from which the LRS material was extruded by hand. **B,C)** Photographs of the length and cross-section of the solid, 1.4 cm-diameter "fiber" extruded from the syringe in B). A US penny is also shown for purposes of scale. **D-F)** Photographs of MRS ink before and after extrusion from a 1.4 cm-diameter syringe; analogous to the LRS shown in A-C.

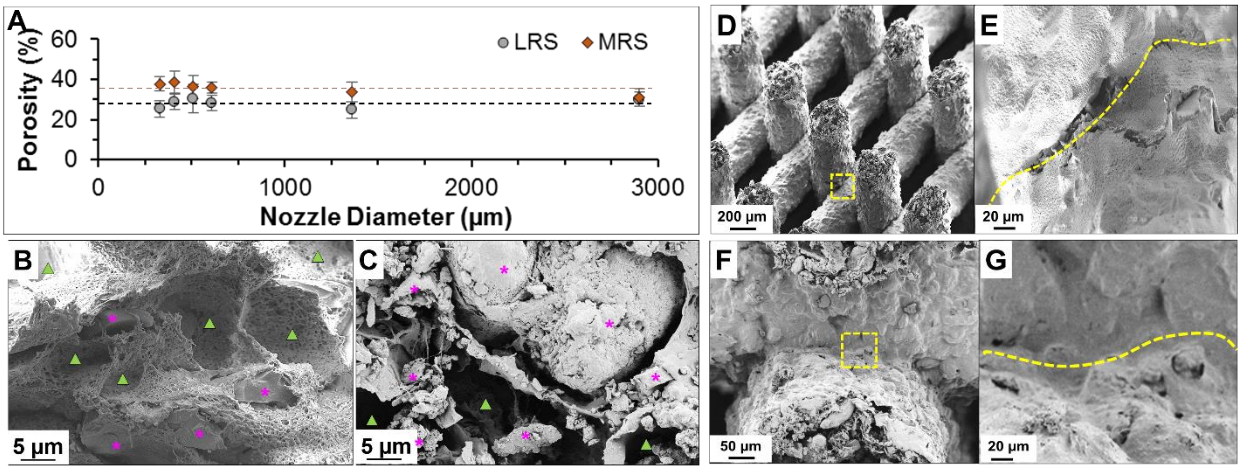

Supplementary Figure 4. A) Extruded LRS and MRS fiber porosity as a function of nozzle diameter. Dotted lines represent aggregated averages for all measured LRS or MRS samples. **B,C)** SEM micrographs further illustrating the interior microstructure of extruded LRS and MRS, respectively. "*" indicate individual LRS or MRS particles; "▲" indicates voids from which LRS or MRS particles were located prior to sample sectioning for imaging and the particles falling out. The PLGA network can be seen throughout both materials. **D-G)** SEM micrographs of the cross-section of 3D-printed LRS (D,F) and MRS (E,G) structures, highlighting interlayer bonding. Due to the near seamless nature of the inter-layer material, a yellow-dashed line (E,G) is provided to further define the boundary between two adjacent layers (Yellow boxes in D,F)

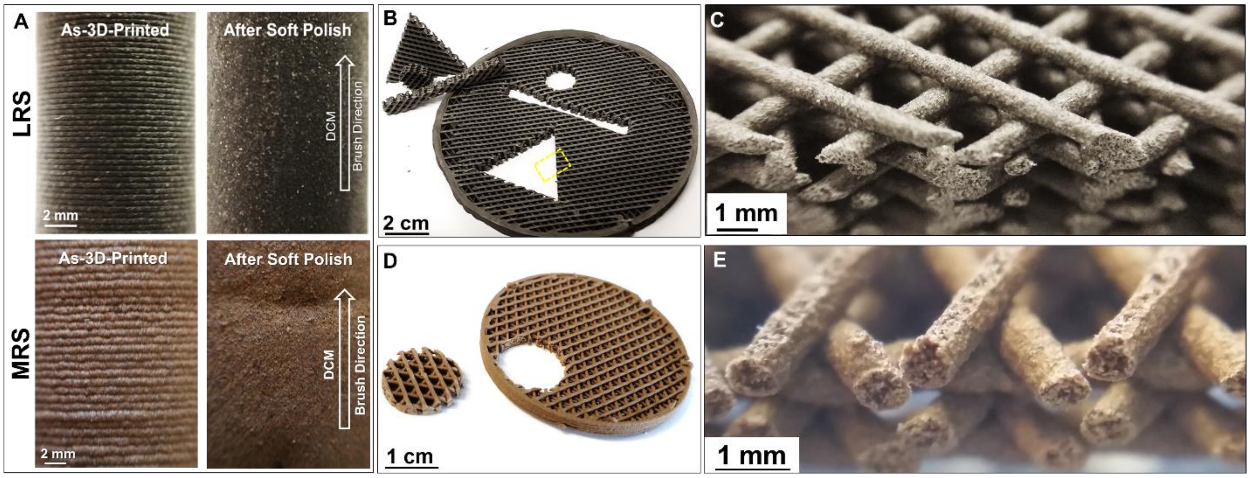

Supplementary Figure 5. A) Close-Up photographs of the external diameter of as-printed (left) LRS (top) and MRS (bottom) cylinders created using 400 and a 600 µm-diameter nozzles, respectively, illustrating innate surface roughness of 3D-printed objects. On right, photographs of the exterior of the after soft-polishing using DCM, which removes the ridges that previously defined each 3D-printed layer. **B)** Photograph of large-diameter LRS cylinder from which several geometries have been cut using a cylindrical punch (cylinder), stamp (tensile "dog bone"), and razor blade (triangle). **C)** Photograph of the razor-blade cut cross-section (yellow-dashed box in C), highlighting lack of plastic deformation in LRS material near the sectioned interface. **D)** Photograph of 4 cm diameter MRS cylinder, from which a 1 cm-diameter was punched. **E)** Close-up photograph of cut MRS contour.

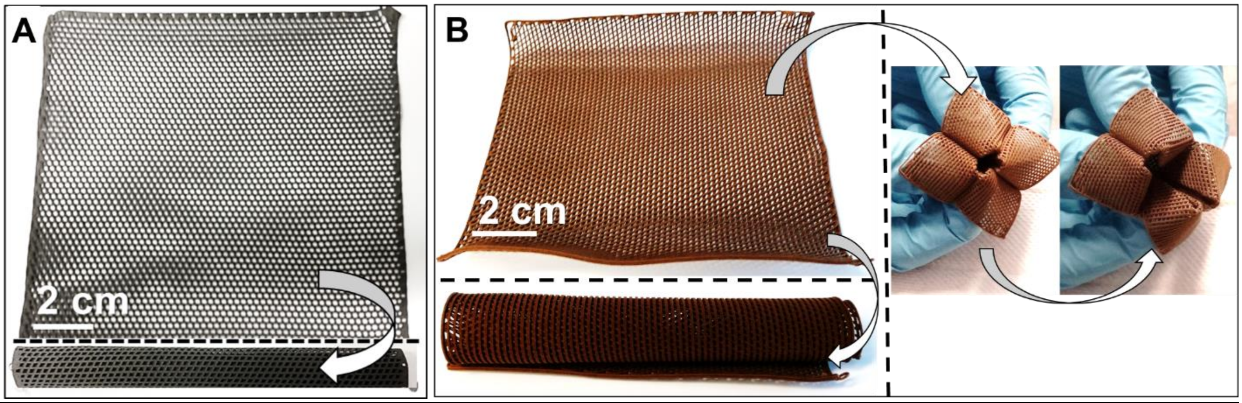

Supplementary Figure 6. A) Photographs of 12x12 cm, three-layer 3D-printed LRS sheet before and after rolling, illustrating flexibility of the as-printed LRS material. **B)** Photograph of 12x12 cm, three-layer 3D-printed MRS sheet before and after rolling and folding.

<u>Supplementary Videos</u>

Supplementary Video 1 – Real Time LRS 3D-Printing: Video illustrating several examples of objects being 3D-printed at rapid speeds from LRS inks. Note rapid drying and fusion of adjacent layers upon material deposition.

Supplementary Video 2. Real Time MRS 3D-Printing: Video illustrating several examples of objects being 3D-printed at rapid speeds from MRS inks. Note similarities to LRS 3D-printing (Supplementary Video 1) and rapid drying and fusion of adjacent layers upon material deposition.

Supplementary Video 3. Real Time 3D-Printing of MRS Sheet and Immediate Post-Printing Handling. Video illustrating the fabrication of a three-layer, 12x12 cm MRS sheet. Immediately after being 3D-printed, it can be removed from the printing platform and handled. Additionally, the video illustrates the flexibility of the sheet.
