# Supplementary material for: Robust and Elastic Lunar and Martian Structures from 3D-Printed Regolith Inks
Source: Sci Rep. 2017 Mar 20;7:44931. doi: 10.1038/srep44931 (PMC5357966; doi:10.1038/srep44931)
Supplement: Supplementary Dataset 1 [file srep44931-s2.doc]

|  | **SiO_2_** | **Al_2_O3** | **Fe_2_O_3_** | **FeO** | **TiO_2_** | **CaO** | **Na_2_O** |
| --- | --- | --- | --- | --- | --- | --- | --- |
| **JSC-1A** | 46 | 15.75 | 12.2 | 8.17 | 1.7 | 9.9 | 2.8 |
| **JSC MARS-1A** | 40 | 22 | 11 | 3 | 3.5 | 5.5 | 2 |
